# Bridging the Care Gap: Family Support for Adults Living with Diabetes Mellitus in Limpopo Province, South Africa

**DOI:** 10.3390/ijerph23040443

**Published:** 2026-03-31

**Authors:** Selina Motsharine-Ndou, Lina Sebolaisi Hlahla, Dorah Ursula Ramathuba

**Affiliations:** 1 Department of Advanced Nursing Science, University Road, University of Venda, Thohoyandou 0950, South Africa; dorah.ramathuba@univen.ac.za; 2 School of Nursing, Faculty of Health Science, University of the Free State, Bloemfontein 9301, South Africa; hlahlals@ufs.ac.za

**Keywords:** adults, diabetes mellitus, family, support

## Abstract

**Highlights:**

**Public health relevance—How does this work relate to a public health issue?**

- Diabetes mellitus is a major public burden in South Africa, specifically in the rural communities where there are resource limitations.
- The study highlights that family support is critical, but not fully utilized, in diabetes mellitus self-management support.

**Public health significance—Why is this work of significance to public health?**

- Disparities in support of patient-related care, such as blood glucose monitoring, foot care, and eye care, reflect systemic inequities in access to resources and health.
- The involvement of the family members directly impacts self-care and the prevention of complications related to diabetes.

**Public health implications—What are the key implications or messages for practitioners, policy makers, and/or researchers in public health?**

- Public health strategies should incorporate family education into primary healthcare services to improve diabetes outcomes in rural communities.
- Policy makers should strengthen family-centred diabetes programs and enhance access to fundamental self-management resources at the community level.

**Abstract:**

The rising prevalence of diabetes mellitus in South Africa presents a critical public health challenge. While the clinical definition involves persistent hyperglycaemia due to insulin irregularities, the daily reality of the disease is deeply rooted in the domestic sphere. Because diabetes often affects multiple family members, the household becomes the primary site of intervention. This study aimed to explore and describe the types of support provided by family members to adult patients with diabetes mellitus in the Mopani and Vhembe districts of Limpopo province, South Africa. A qualitative, exploratory, descriptive, and contextual approach was used to investigate family members’ support for patients with diabetes. A non-probability, purposive sampling was used to select participants. Semi-structured interviews were used for data collection, and Tesch’s open coding method was used for data analysis. Patient support by family members included lifestyle modification support and emotional support. Family support for individuals living with diabetes mellitus is vital. Time and again, family plays a key role in supporting a member living with diabetes mellitus.

## 1. Introduction

Diabetes mellitus is a group of common endocrine disorders characterized by persistent hyperglycaemia resulting from insufficient insulin secretion, impaired insulin action, or a combination of both [1,2]. The global prevalence of diabetes mellitus is increasing steadily, with the most pronounced growth occurring in low- and middle-income countries [3]. In South Africa, type 2 diabetes mellitus is highly prevalent and frequently occurs within families, placing a substantial health burden not only on affected individuals but also on their households.

The effective management of diabetes mellitus largely relies on patients’ engagement in daily self-care activities, including blood glucose monitoring, medication adherence, dietary modification, and regular physical activity. However, family involvement has been shown to play a critical role in supporting these self-management practices and improving health outcomes [4]. Although many individuals living with diabetes mellitus value family involvement in their care, the chronic nature of the condition and its long-term treatment may impose emotional, physical, and financial challenges on family members. Evidence suggests that actively involving family members in diabetes mellitus management and equipping them with appropriate knowledge and skills can significantly improve diabetes-related outcomes [5]. Consequently, family support remains a cornerstone of effective diabetes care.

Family support contributes to both the physical and psychological well-being of patients by fostering comfort, self-confidence, and a sense of being valued and cared for. These psychosocial benefits may enhance patients’ motivation to engage in self-care behaviors, such as regular self-monitoring of blood glucose levels [6]. Previous studies have demonstrated a positive association between family support and adherence to self-management behaviors, including medication compliance and glycemic monitoring [6,7]. A study conducted in rural China demonstrated the critical role that families play in the self-management of patients’ diabetes journey [8]. Similarly, a study conducted in Nigeria indicated that family support correlates with improved medication adherence and, consequently, superior glycemic control [9]. However, a study conducted in Egypt reported no significant relationship between family support, self-care practices, and glycemic control, underscoring the need for further research to better understand the nature and adequacy of family support required by patients with diabetes mellitus [10].

Strong family support also encourages patients to assume greater responsibility for their health while providing emotional reassurance during stressful periods [9]. Family members often assist by facilitating routine health check-ups, ensuring timely medication intake, preparing appropriate meals, and encouraging physical activity [7]. Despite the recognized benefits of such involvement, family support for individuals living with chronic conditions, including diabetes mellitus, remains insufficient in some settings. This inadequacy is frequently attributed to limited knowledge, awareness, or understanding of the disease among family members [11].

Previous research conducted in Indonesia has identified four key dimensions of family support for patients living with type 2 diabetes mellitus: emotional, appreciation, instrumental, and informational support [12,13]. Emotional support includes expressions of empathy, trust, love, understanding, and encouragement, while appreciation support involves praise, recognition, and constructive guidance. Instrumental support refers to tangible assistance, such as financial support, home-based care, appropriate nutrition, and facilitation of family discussions related to health needs [12,13]. Informational support encompasses advice and guidance on diabetic diets, physical activity, recognition of complications, and adherence to prescribed treatment regimens [12,13]. Collectively, these forms of support have been shown to enhance psychosocial well-being, motivation, and self-esteem among patients living with diabetes mellitus.

Family support is therefore widely recognized as an essential component of comprehensive diabetes management. This study aimed to explore and describe the forms of support provided by family members to adult patients living with diabetes mellitus in the Vhembe and Mopani districts of Limpopo Province, South Africa.

## 2. Materials and Methods

### 2.1. Research Design

A qualitative, exploratory, descriptive design was used to help the authors gain insight, add knowledge, and describe how family members support their relatives living with diabetes mellitus in this study [14]. The study was conducted in the Mopani and Vhembe districts of Limpopo Province, South Africa. Data were collected in the homes where family members live with patients with diabetes mellitus.

### 2.2. Study Setting

The study was conducted in the Mopani and Vhembe districts of Limpopo Province, South Africa. Data were collected in the homes where family members live with patients with diabetes mellitus. During data collection, patients were asked to provide contact details of family members who support them in the management of diabetes mellitus, in preparation for interviewing them at home after obtaining informed consent to participate in the study.

### 2.3. Population and Sampling

Family members who stay and provide support to their members who are living with diabetes mellitus were requested to form part of the study. A non-probability, purposive sampling was used to select the participants. A sample of 14 female family members who stayed and assisted patients diagnosed with diabetes mellitus with self-care management was included in the study until data saturation.

The exclusion of male participants in this study was not intentional; rather, it resulted from the availability of female participants during data collection. Although the study attempted to include both male and female volunteers who matched the inclusion criteria, men were either unavailable during recruiting or declined to participate. The women who participated met the inclusion criteria and provided rich, detailed narratives for the research study. Their narratives were sufficient to fully explore the phenomena under study.

### 2.4. Inclusion and Exclusion Criteria

Participants were included if they were 18 years of age or older, had stayed with the patient in the past 6 months, and were willing to participate in the study. Excluded were family members aged 18 or older who were not willing to participate in the study.

### 2.5. Data Collection Procedures

Semi-structured interviews were used to collect data. A central question (may you kindly share with me the type of support you provide to the family member living with diabetes mellitus) was used to direct the data collection process, followed by predetermined open-ended questions and probing questions to gather additional information. Data were first collected in Mopani using Xitsonga and subsequently in Vhembe district using Tshivenda, the local languages of the districts, and then later translated to English. Additionally, field notes were taken, and key issues and observations of nonverbal cues were documented. Interviews lasted approximately 30–45 min and continued until data saturation was reached. While data saturation was reached at the 14th participant, the authors conducted further interviews to ensure the robustness of the findings. Subsequent interviews yielded no novel themes or insights, thereby confirming that empirical saturation had been achieved and that the established thematic framework was comprehensive. To ensure precise data capture, credibility, and prevent data loss, an audio tape recorder was used, as agreed with the participants. Interviews were conducted after obtaining written and verbally informed consent from participants. Access to raw data was restricted to the authors only.

### 2.6. Data Analysis

All interviews were transcribed verbatim in Xitsonga and Tshivenda. Then translated into English by a language expert. The translated data were coded manually with assistance from the independent coder, a PhD holder and Professor at the University of South Africa.

The transcribed data was analysed by the authors using Tesch’s eight steps of coding method [15]

1. Get a sense of the whole

The authors reviewed the transcripts and identified key ideas to better understand the interview. Every transcript was reviewed to grasp the information shared by the participants. The authors understood the interviews and analysed them into significant concepts.

2. Choose one interesting interview

The authors selected an interesting interview and examined its deeper significance while writing down notes in the margins

3. Similar topics clustered together

The authors grouped related topics together and organized them into main and unique topic categories.

4. Go back to the data

The authors reviewed a list of subjects and returned to the data; the subjects were represented by codes. Placing the codes alongside the relevant sections of the text allowed the authors to determine whether new categories and codes continued to appear.

5. Identification of the most descriptive wording for topics

The authors pinpointed the most descriptive language for the subjects and transformed them into key themes. Key themes were condensed by clustering similar subjects. Connections were established among the key themes to illustrate their interrelations

6. Abbreviation of categories

Abbreviations for every category were generated, and the codes were sorted categorically to guarantee no repetition.

7. Similar data assembled

The authors performed an initial analysis by gathering the data for each category in a single location

8. Data recording

The current data was documented and securely kept on a password-protected device. The authors outlined the main themes and sub-themes before forwarding the raw data to a separate coder who assisted with the coding process [15]. Subsequently, the authors and the independent coder concurred on the established primary themes, themes, and subthemes.

The main 2 main themes that came out of this study were Lifestyle Modification Support and Emotional Support.

#### Measures to Ensure Trustworthiness

Trustworthiness was ensured through credibility, prolonged engagement, confirmability, and independent coding [15] as in Table 1.

### 2.7. Ethical Considerations

Ethical clearance was obtained from the University of Venda Research Ethics Committee (reference number SHS/20/PDC/39/2110), and permission was granted by the University of Venda Higher Degree Committee, the Department of Health, Limpopo Province research committee, and the Vhembe and Mopani district research committee, as well as from the selected community health centres. Written and verbal consent to participate in the study and to be recorded during the interview was obtained from participants.

## 3. Results

### 3.1. The Demographic Data

Table 1 presents the demographic data of family members. Family members who assisted individuals living with diabetes mellitus were interviewed; predominantly females aged 29 to 57 years.

### 3.2. Themes and Subthemes

Table 2 presents the demographic data of family members who assisted individuals living with diabetes mellitus who were predominantly females aged 29 to 57 years.

Table 3 presents the themes and their associated subthemes.

The findings of this study revealed two main themes describing the types of support family members provide to adult patients with diabetes mellitus: Lifestyle Modification Support and Emotional Support. Each theme is presented with its associated subthemes and supported by verbatim quotations from participants.

#### 3.2.1. Theme 1: Lifestyle Modification Support

Family members played a significant role in supporting patients in implementing and maintaining lifestyle modifications essential to effective diabetes mellitus management. This support was evident in blood glucose monitoring, dietary management, medication adherence, physical care practices, and weight management.

Support with Blood Glucose Monitoring

Some family members assisted patients with monitoring blood glucose levels at home, particularly when patients were unwell. Support included performing blood glucose tests, recording results, and responding to abnormal readings as advised by health care professionals. One participant stated, “*My mother monitors her blood glucose levels on her own*. *I only monitor her blood glucose when she is not well… If her blood glucose level is high*, *I advise her to drink water to help lower it to a normal level.*” (FM2, 32 years)

Another family member reported: “*I monitor my mother’s blood glucose levels once a day*, *or when she looks ill*. *I record her blood glucose levels in the diary as advised by the nurses*.” (FM3, 29 years)

However, other family members reported challenges in providing this support due to financial constraints and lack of access to blood glucose monitoring equipment: “*It is not possible to buy blood glucose monitors and strips regularly because they are too expensive… The government should assist by providing us with machines and test strips*.” (FM4, 42 years)

These findings indicate that although some family members supported blood glucose monitoring, others were unable to do so due to limited resources, such as glucose monitoring machines.

Dietary Management Support

Dietary support was provided through the preparation and encouragement of healthy meals as recommended by a healthcare professional. Some family members reported modifying cooking methods and limiting sugar and salt intake: “*I boil or roast most of the food he eats. I don’t add too much salt… I give him brown bread and soft porridge in the morning.*” (FM4, 42 years)

The participant further gave a report on how she supports her husband, who is diabetic, in making sure he eats: “*we also have started a garden at home so that we get vegetables to assist his health*.” (FM4, 42 years)

Despite this, other participants reported difficulty in supporting dietary management due to financial limitations: “*Most of the things needed for a healthy diet require money… If the Department of Health can provide food parcels, it will be of great help.*” (FM13, 57 years)

These findings suggest that while dietary support was commonly provided, socioeconomic challenges hindered consistent adherence to recommended diets.

Medication Adherence Support

Family members supported patients by reminding them to take medication and ensuring adherence to prescribed schedules. One participant explained: “*I make sure that before my husband eats in the morning; he takes his medication… He takes it at 7h00 and 19h00.*” (FM4, 42 years)

Others indicated that patients were largely independent but still monitored: “*I monitor if my mother is taking her medications, but she usually takes them on time without assistance*.” (FM2, 34 years)

This demonstrates that family involvement contributed positively to medication adherence.

Physical Care Support

Physical care support included assistance with exercise, foot care, and eye care. Family members gave accounts of how they supported their loved ones with diabetes in relation to physical support.

Exercise Support

Family members encouraged and assisted patients to engage in physical activity appropriate to their abilities. One participant said that: “*We assist her to exercise her arms*, *hands*, *and chest according to her ability*.” (FM3, 29 years)

Another participant stated: “*I always encourage my husband to use the bicycle for exercise. He cycles for an hour every day. I also encourage him to do walking exercises*” (FM4, 42 years)

These findings indicate active family involvement in promoting physical activity by encouraging diabetic patients to exercise.

Foot Care Support

Some family members demonstrated adequate knowledge and actively assisted with foot care practices. This was confirmed by a participant who said that: “*I help my husband wash his feet*, *dry between the toes*, *apply lotion*, *and check for wounds every day*.” (FM8, 42 years)

However, other participants lacked knowledge regarding foot care: “*There is nothing I can do to assist with foot care because my husband doesn’t complain of a foot problem*.” (FM4, 42 years)

This highlights variability in family members’ understanding and implementation of foot care support.

Eye Care Support

Most family members reported limited knowledge regarding eye care practices, particularly preventive measures such as routine eye examinations. Participant 38 reported her limited knowledge by saying, “*I do not know how to assist my mother with eye care because she has never had eye problems*.” (FM6, 38 years)

Some believed eye care was only necessary when problems occurred: “*I didn’t know that I should take her for an eye examination*.” (FM7, 37 years)

These findings indicate inadequate awareness of eye care among family members.

Weight Management Support

Family members supported weight management by encouraging exercise and physical activity to promote weight loss. The family members gave the following accounts in relation to weight management support: “*I climb stairs with her and do exercises to help her lose weight and improve circulation*.” (FM1, 32 years)

Others encouraged regular movement: “*I always tell him that to lose weight, he should exercise every day for at least 45 min.*” (FM5, 48 years)

The quotes from family members reflect recognition of weight control and weight support management as important components of diabetes management.

#### 3.2.2. Theme 2: Emotional Support

Family members provided emotional and psychological support to help patients cope with the demands of living with diabetes mellitus. This support was expressed through empathy, encouragement, and stress reduction.

Emotional Encouragement and Empathy

Participants described providing reassurance, compassion, and a supportive environment to support their family members with diabetes. The claims below serve as evidence of what the family members said.

One participant reported providing psychological support to their mother by praying with her, reassuring her, and encouraging her not to worry excessively about her condition, emphasizing that she will be fine: “*I pray for my mother. I also constantly tell her not to worry or be stressed. I believe she will be well and I tell her that she will be well. I also tell her that her condition is manageable if she complies with the advice from the healthcare professionals.*” (FM3, 29 years)

Another participant explained how empathy assists their family members in managing the disease by saying, “*I am always available when he needs me. I make sure I listen to him when he talks, especially about his condition and needs related to his condition*…*The emotional support we give him helps him manage his condition and cope better. His coping skills and resilience are also improved, which makes him more determined to manage the disease*” (FM5, 48 years)

The quotes from the family members demonstrate that family members understand that emotional encouragement and showing empathy go a long way in showing support to family members with Diabetes Mellitus.

Psychological Coping and Stress Management Support

Participants gave descriptions of how they assisted their family members in coping and managing stress related to their condition. Family members encouraged patients to express emotions and discuss stressors: “*I encourage my mother to express her emotions, as it helps her release emotional tensions. I also advise her on how to share how she needs support from people in her life. I tell her to be open with us about anything troubling her related to her condition.*” (FM6, 38 years)

Participant 10 described supporting her sister by saying, “*I encourage my sister to minimise stress related to her illness and by offering spiritual support through prayer, expressing a belief that through faith in God and confidence in the effectiveness of medication would contribute to her recovery*” (FM10, 56 years)

These findings indicate that emotional support from family members plays a crucial role in enhancing patients’ psychological well-being and their ability to cope effectively with their condition.

## 4. Discussion

The findings of this study reveal that family members primarily support patients with diabetes mellitus by assisting with lifestyle modification strategies. This includes assisting with blood glucose monitoring, promoting healthy dietary habits, encouraging adherence to prescribed medication, supporting physical activity, and managing weight. In addition to practical support, family members also provide emotional encouragement and empathy, as well as assistance with psychological coping and stress management.

Family members reported supporting diabetic patients by monitoring their blood glucose levels. However, limited access to blood glucose monitoring equipment emerged as a significant barrier, leading to generally poor family support for it. This finding is consistent with the following studies conducted in resource-limited settings: A study conducted in low-resource settings in Uganda and Ethiopia found that a lack of access to glucometers or glucose strips was a barrier to regular blood glucose monitoring, even with family support [16,17].

The study found that some family members supported patients with diabetes mellitus by preparing and serving recommended healthy meals as advised by healthcare professionals. The literature indicates that male patients are more likely to receive dietary and nutritional support from their wives, who often have greater knowledge of healthy diets and practical cooking skills [18]. However, not all family members could provide adequate dietary support, as financial constraints prevented them from purchasing healthy food. The findings concur with [19], who indicated that socio-economic constraints pose a challenge to affording a healthy diabetic diet. A healthy diet remains essential in controlling blood glucose levels among patients living with diabetes mellitus.

The findings further demonstrated that family members supported patients by reminding them to take diabetes mellitus medications and ensuring adherence to prescribed medication regimens. Similar findings were reported in a study conducted in Ethiopia, where family members played a significant role in providing patients with medication [20]. Patients who received family support were three times more likely to adhere to their treatment regimens than those who did not, highlighting the critical role of family involvement in medication adherence [21].

Physical care support was also evident, with some family members actively encouraging patients to engage in exercise, foot care, and eye care. Family encouragement appears to play an important role in promoting adherence to recommended physical activity. The finding shows that patients are encouraged and assisted in performing cycling, walking, and arm exercises tailored to their abilities. These findings are supported by previous studies indicating that family members motivate patients to participate in physical activity and, in some cases, accompany them during walks as part of lifestyle modification efforts [22,23].

Family encouragement appears to play an important role in promoting adherence to recommended physical activity.

Foot care emerged as another area of family support, although knowledge and involvement varied. Proper foot care for patients living with diabetes mellitus includes washing and thoroughly drying the feet, applying lotion while avoiding the area between the toes, wearing appropriate footwear, and conducting daily inspections for cuts, sores, swelling, or infections [24]. Adequate foot care is essential in preventing complications such as diabetic foot ulcers and lower-limb amputations. While some family members supported patients in managing foot care, others lacked the necessary knowledge, resulting in limited or no support. Similar findings have been reported in previous studies, in which family members provided advice and assistance with foot care, whereas others did not do adequate foot care due to insufficient information [25,26,27].

The study further revealed inadequate knowledge among family members regarding eye care. Many participants were unaware of preventive eye care practices, such as routine annual eye examinations, which are crucial for the early detection and management of diabetes-related eye complications. Additional protective measures, including wearing sunglasses and using umbrellas to shield the eyes from sunlight, were also poorly understood. These findings are consistent with studies conducted in Ethiopia, which reported low utilization of eye care services among diabetic patients [28]. In agreement, studies from the United States of America reported higher non-adherence to recommended eye care practices related to financial constraints [29,30].

Furthermore, family support related to eye and foot care was generally inadequate due to insufficient knowledge. While some studies have reported adequate family support for eye care among certain patients, others have highlighted gaps in support [31,32]. The consequences of poor diabetes management, particularly diabetic foot complications, can be severe, including amputations, reduced functional ability, increased financial burden, and diminished quality of life. Educating family members about foot care has been shown to enhance patients’ self-care behaviors, as family members are closely involved in daily care [33].

Weight control support was identified as an important lifestyle modification strategy in diabetes management. Weight control involves achieving and maintaining a healthy body mass index through balanced dietary intake and regular physical activity [34,35]. In this study, family members regarded weight management as vital and supported patients by encouraging light physical activities according to their abilities. Although some patients were reluctant to exercise, family members persisted in promoting awareness of the importance of weight loss. These findings are supported by previous research indicating that family members significantly influence patients’ daily behaviors and frequently encourage activities such as walking and stair climbing to maintain a healthy body weight [36].

Diabetes mellitus is a chronic condition that affects not only physical health but also emotional and psychological well-being. Patients may experience diabetes-related distress, depression, anxiety, and food-related concerns [37]. The findings of this study indicate that family members are concerned about patients’ emotional well-being and provide support to help them cope, thereby promoting effective diabetes management.

Emotional distress is common in patients living with diabetes mellitus. According to reports, people living with diabetes suffer from emotional distress or mental health problems [38,39]. This study identified that emotional support was another critical component of family involvement. Emotional support, which encompasses empathy, compassion, and genuine care, is a fundamental human need [40]. Family members provided emotional encouragement, enabling patients to cope with anxiety, stress, and the psychological burden associated with diabetes mellitus. Such support contributed to patients’ resilience and strengthened their ability to manage the condition effectively. This is consistent with other studies [41,42,43] that found that enhancing emotional support is a useful strategy for reducing the burden of psychological distress and encouraging better patient self-care.

**Implications to Practice**

These findings emphasize the need for health care professionals to adopt a holistic approach to the management of adult patients with diabetes mellitus. They need to involve family members in patient care. They must educate them on how to support their loved ones living with diabetes mellitus. When family members know the complications they can anticipate, they can better support their loved ones. Appropriate guidance from health care professionals may enhance family support and reduce the risk of unnecessary diabetic complications.

The health care professionals working in health centres should embrace a family-centred approach to diabetes management. Providing health education to family members alongside patients may enable them to provide the needed support, particularly in areas where the study identified gaps, such as blood glucose monitoring, foot care, and eye care. Family members may be empowered, and patient outcomes may be enhanced by organized health education programs that use practical examples and culturally relevant information.

Furthermore, addressing socioeconomic barriers such as limited access to monitoring equipment and healthy food requires collaboration between healthcare providers and policymakers. Strengthening community-based support systems and improving access to essential diabetes management resources may reduce the burden on families and enhance self-care practices.

**Limitations of the study**

The sample’s homogeneity, consisting solely of women, is a major limitation of this study. Although recruitment attempts targeted all genders, male invitees either refused to participate or did not attend the data collection sessions, limiting the generalisability of the findings to the larger population.

## 5. Conclusions

Overall, this study underscores that effective diabetic self-care management is significantly influenced by family support. There are still gaps in blood glucose monitoring and preventive care practices, despite family members providing significant support with dietary management, medication adherence, physical exercise, and mental well-being. Patients with diabetes mellitus may have better diabetes outcomes and a higher quality of life if health care providers’ interventions are used to strengthen family education and involvement.

## Figures and Tables

**Table 1:** Measures to ensure trustworthiness.

Strategy	Criteria	Applicability
Credibility	Prolonged engagement	Data were collected for 4 months until data saturation was reached to gain an in-depth understanding of the phenomenon.
	Member Checking	The authors replayed the audio tapes to the participants to verify the overall interpretation and meaning of the findings
Transferebility	Data Saturation	Data was collected until saturation was reached
	Detailed description	Authors provided in-depth description of the context that influenced the research process and its findings
Confirmability	Audit trail	written consent forms, transcripts, a voice recorder, and field notes that were used to record the interview, to avoid biases or perspectives, were kept safe after data analysis
	Triangulation	Authors used different data collection tools, such as a voice recorder & field notes, during the interview
Dependability	Dense Description	A purposive sampling technique where participants were chosen based on their knowledge of the phenomenon under investigation
	Coding	After data analysis, themes and subthemes emerged, and a consensus was reached with the independent coder.

**Table 2:** Demographic data of the family member.

Family Members		
Participants Code	Age	Gender
FM 1	32	F
FM 2	34	F
FM 3	29	F
FM 4	42	F
FM 5	48	F
FM 6	49	F
FM 7	34	F
FM 8	42	F
FM 9	56	F
FM 10	56	F
FM 11	32	F
FM 12	55	F
FM 13	57	F
FM 14	43	F

**Table 3:** Theme and Subthemes.

Theme	Subtheme
Lifestyle Modification Support	Support with Blood glucose monitoring
	Dietary management support
	Medication adherence support
	Physical care support (exercise, foot care, eye care)
	Weight control support
Emotional Support	Emotional encouragement and empathy
	Psychological coping and stress management support

## Data Availability

The data presented in this study are available upon request from the corresponding author due to ethical considerations.
